# Cytotoxicity and chemical composition of women's personal PM_2.5_ exposures from rural China†

**DOI:** 10.1039/d1ea00022e

**Published:** 2021-07-27

**Authors:** Alexandra Lai, Jill Baumgartner, James J. Schauer, Yinon Rudich, Michal Pardo

**Affiliations:** Department of Earth and Planetary Sciences, Weizmann Institute of Science Rehovot Israel michal.levin@weizmann.ac.il; Institute for Health and Social Policy, Department of Epidemiology, Biostatistics, and Occupational Health, McGill University Montreal Quebec Canada; Environmental Chemistry & Technology Program, University of Wisconsin–Madison Madison WI USA

## Abstract

Personal exposure PM samples aid in determining the sources and chemical composition of real-world exposures, particularly in settings with household air pollution. However, their use in toxicological research is limited, despite uncertainty regarding health effects in these settings and evidence of differential toxicity among PM_2.5_ sources and components. This study used women's PM_2.5_ exposure samples collected using personal exposure monitoring in rural villages in three Chinese provinces (Beijing, Shanxi, and Sichuan) during summer and winter. Water-soluble organic carbon, ions, elements, and organic tracers (*e.g.* levoglucosan and polycyclic aromatic hydrocarbons [PAHs]) were quantified in water and organic PM_2.5_ extracts. Human lung epithelial cells (A549) were exposed to the extracts. Cell death, reactive oxygen species (ROS), and gene expression were measured. Biomass burning contributions were higher in Sichuan samples than in Beijing or Shanxi. Some PM characteristics (total PAHs and coal combustion source contributions) and biological effects of organic extract exposures (cell death, ROS, and cytokine gene expression) shared a common trend of higher levels and effects in winter than in summer for Shanxi and Beijing but no seasonal differences in Sichuan. Modulation of phase I/AhR-related genes (cyp1a1 and cyp1b1) and phase II/oxidative stress-related genes (HO-1, SOD1/2, NQO-1, and catalase) was either low or insignificant, without clear trends between samples. No significant cell death or ROS production was observed for water extract treatments among all sites and seasons, even at possible higher concentrations tested. These results support organic components, particularly PAHs, as essential drivers of biological effects, which is consistent with some other evidence from ambient PM_2.5_.

## Abstract 2

Direct measurement with personal samplers captures the chemical complexity of PM_2.5_ exposures better than fixed monitors. To investigate biological effects, lung cells were exposed to extracts of exposure PM_2.5_ samples.

## Graphical abstract

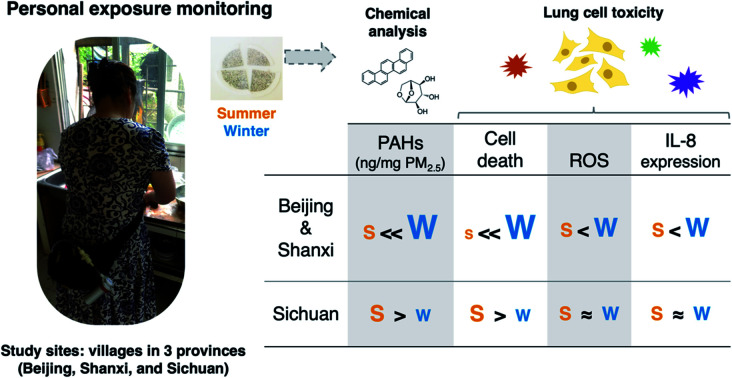

**Environmental significance**

Atmospheric particulate matter (PM) is typically monitored and regulated in terms of total mass, yet there is evidence that sources and chemical components of PM may contribute differentially to observed health effects. Numerous natural and anthropogenic sources contribute to air pollution exposure – including household air pollution, particularly for the over three billion people worldwide using solid fuels like coal and wood for cooking and heating. Direct exposure measurements using portable samplers in the field capture this complex mixture. However, PM exposure samples are rarely used in laboratory studies of PM health effects. Here, we collected PM_2.5_ exposure samples in villages in China and assessed their effects on lung cells *in vitro* with respect to varying chemical composition, season, and location.

## Introduction

Particulate air pollution is the leading environmental health risk factor globally, responsible for an estimated 6.4 million premature deaths annually according to the 2019 Global Burden of Disease study.^1^ Decades of epidemiological research have established causal relationships between fine particulate matter (PM_2.5_) and overall mortality and a range of health impacts including cardiopulmonary outcomes.^2^ Current research from cellular and molecular approaches to epidemiologic studies aims to reduce uncertainty in these relationships and understand the underlying mechanisms and how these may be affected by PM source and physicochemical characteristics.^3^

Exposure assessment is a key component of estimating exposure–response relationships.^3^ Most epidemiological studies have used ambient PM measurements from a central study site or routine air quality monitoring as a proxy for exposures.^2^ However, exposures are also influenced by multiple variables such as indoor environments, time–activity patterns, and small-scale spatial heterogeneity.^4^ The resulting exposure error tends to underestimate health effects associations in epidemiological studies.^2^ To more accurately measure personal exposures, small wearable samplers and sensors have been developed to measure real-time PM concentrations and/or collect filter samples for gravimetric and chemical analysis and are increasingly common in air pollution epidemiology research.^3,4^ Studies comparing concurrent outdoor and personal exposure samples report potentially important differences: for example, in a study of healthy adults in Hong Kong, fractional exhaled NO (a marker of airway inflammation) was positively associated with personal exposure to PM_2.5_, but not with ambient PM_2.5_.^5^

Indoor air pollution sources further complicate exposure assessment. Over three billion people globally rely on solid fuels such as coal and biomass as their household energy sources.^6^ Inefficient combustion of these solid fuels releases pollutants including PM_2.5_ inside the home, creating household air pollution that residents are exposed to. Although the proportion of the global population using solid fuels has decreased in recent years, household air pollution still accounted for 43% of the disease burden attributable to air pollution in 2019.^1^ In these settings, solid fuel combustion is a major, but not the only, source contributing to exposures: paired kitchen area and personal exposure PM_2.5_ measurements are typically only moderately or poorly correlated,^7–9^ and source apportionment analyses report contributions to exposures from non-household sources, such as vehicles and dust.^10^ Direct sampling of PM_2.5_ exposures is therefore uniquely positioned to illustrate the complex mix of sources contributing to total exposure among individuals exposed to household air pollution.

Understanding the causes of observed health effects requires integrating epidemiologic evidence with *in vivo* and *in vitro* laboratory studies. Personal exposure PM sampling is historically less common than area measurements and tended to have less mass than ambient samples: personal samplers operate at 1–4 L min^−1^ on smaller filters, compared to ambient samples operating at 50–100 L min^−1^ on larger filters. Unlike stationary sampling, adding parallel filters and/or samplers is not an option in personal exposure sampling, and battery-powered pumps that are lightweight and quiet enough for personal sampling have limited flow rates. For these reasons, biological analysis has typically not been feasible. However, as personal sampling technology improves and becomes more widely used, the epidemiology studies' large sample sizes can compensate for low mass loadings, as samples can be composited to yield sufficient mass for *in vitro* assays. A few studies have reported *in vitro* analyses of exposure PM samples,^11–17^ but many were in urban settings without reported household use of solid fuels, leaving a knowledge gap in toxicology of PM field samples representing realistic exposures, which are highly relevant to assessments of the health effects of household air pollution.

Here, we analyzed personal exposure PM_2.5_ samples collected in winter and summer from women living in rural or peri-urban villages in three Chinese provinces (Beijing, Shanxi, and Sichuan) with varying degrees of biomass and/or coal use. Exposure PM_2.5_ samples were extracted in water and organic solvents, and chemical components including organic molecular markers, elements, and water-soluble organic carbon and ions were measured. We exposed human lung epithelial cells (A549) to these PM_2.5_ extracts and measured cell death, reactive oxygen species (ROS), and gene expression to evaluate their health impact.

## Methods

### PM_2.5_ sampling

The PM_2.5_ samples used in these experiments were collected as part of two epidemiological studies in China, both of which have been described previously.^8,18,19^ Sichuan samples were collected from female participants in rural villages during a government-sponsored household energy initiative in 2014–2016,^8^ and Shanxi and Beijing samples were collected from male and female participants in rural villages from the multi-province INTERMAP China Prospective study in 2015–2016.^18,19^ At all three sites, subsistence farming was the main occupation of the majority of study participants (>70%) and study participants used solid fuels for household cooking and heating.^8,19^ Biomass and LPG were used for cooking at all three sites, but heating fuels differed: coal was generally used for heating in households at the Shanxi and Beijing sites, while households at the Sichuan site used biomass. The Sichuan study site was the most rural, whereas villages at the Shanxi and Beijing sites are in the process of becoming more peri-urban, and the Beijing site in particular was more influenced by nearby sources. Informed consent was obtained from all participants. Study protocols were approved by ethical review boards at the investigator institutions for each study (Sichuan: McGill University, Tsinghua University, and University of Wisconsin–Madison; Beijing and Shanxi: McGill University, Imperial College London, Peking University, Tsinghua University, and Fu Wai Hospital).

Sampling was conducted using 37 mm Zefluor PTFE filters (Pall Life Sciences, USA) using Harvard personal environmental monitors (Mesa Labs, USA)^20^ with personal sampling pumps (Apex Pro and TUFF™, Casella Inc., USA) operating at 1.8 lpm.^8,19^ Study participants carried the samplers in waistpacks or placed samplers nearby if necessary (*e.g.* during bathing and sleeping). Pedometers were also included in the waistpacks to monitor compliance. Each filter was sampled for 24 hours (Beijing and Shanxi) or 48 hours (Sichuan). Filters were weighed before and after sampling using an automated microbalance in a temperature- and humidity-controlled environment.^8^

In order to obtain sufficient mass for both chemical analyses and biological assays, multiple randomly selected filters from each sampling location were composited to create the samples discussed here. For each site (Beijing, Shanxi, and Sichuan) in each season (summer and winter), a composite was created that consisted of 20–40 filters, depending on mass loading. For consistency across study sites, only samples from female participants were included in composites. In each composite, filters were cut in half, and halves were combined into paired identical composites for organic and water extraction. Details of mass and filters in each composite are available in the ESI (Table S1†).

### Chemical characterization of PM_2.5_

Collection and chemical analysis of PM_2.5_ samples have been described in detail previously.^8,21,22^ Briefly, for water extracts, filters were submerged in 10–15 mL (depending on number of filters) of ultrapure water, sonicated for 30 minutes, shaken on a shaker table for 6 hours, and sonicated again for 30 min. After removing filter pieces, aliquots were used to measure water-soluble organic carbon (WSOC), inorganic ions, and total element concentrations. WSOC was measured using a total organic carbon analyzer (M9 TOC Analyzer, Sievers/GE). Ions (Na^+^, K^+^, NH_4_^+^, Cl^−^, SO_4_^2−^, and NO_3_^−^) were measured using ion chromatography (Dionex ICS 1100 and 2100, Thermo Fisher Scientific, USA). Elements were measured using high-resolution inductively-coupled plasma mass spectrometry (Thermo-Finnigan Element 2, Thermo Fisher Scientific, USA) following microwave digestion with ultrapure HNO_3_.

For organic extracts, filters were extracted by Soxhlet in 50 : 50 dichloromethane/acetone, filtered through a 0.22 μm PTFE syringe filter, and concentrated by rotary evaporation and under N_2_ gas. An aliquot of each extract was methylated, and organic compounds were measured using GC/MS (GC-6980, quadrupole MS-5973, Agilent Technologies, USA) as described previously.^23^ Water and organic extracts were stored at −20 °C between analyses.

### Cell culture and exposure to PM_2.5_ extracts

Alveolar epithelial cells from the human lung adenocarcinoma cell line A549 (CCL-185, ATCC) were grown in RPMI-1640 medium (Gibco, Thermo Fisher Scientific, USA) supplemented with 10% fetal bovine serum and 1% penicillin/streptomycin (Biological Industries, Beit Ha-Emek, Israel). A549 cells are one of the most commonly used cell lines for *in vitro* research on the mechanisms of PM health effects,^24,25^ and studies comparing A549 and non-cancerous human lung epithelial cells have observed similar trends in toxicities of different PM samples.^26,27^ The cells were grown in a humidified atmosphere with 5% CO_2_ at 37 °C.

Cells were seeded in 12-well plates (Corning, USA) on the day before exposure to PM at a concentration of 150 000 cells per mL. For biological analyses, unmethylated organic extracts were transferred into DMSO, with a N_2_ flow to evaporate the remaining traces of dichloromethane. Stock solutions of water extracts and organic PM_2.5_ extracts were diluted to the stated exposure concentrations in sterile ultrapure water buffered with a salts–glucose medium (SGM) consisting of 50 mM HEPES, 100 mM NaCl, 5 mM KCl, 2 mM CaCl_2_, and 5 mM glucose at pH 7.2. Organic extract solutions for cell exposures had a final DMSO concentration of 5%. For blank controls, cells were exposed to solutions with the same SGM buffer concentrations and, for the controls corresponding to the organic extracts, 5% DMSO. For cytotoxicity experiments, and cells were exposed for 24 and 5 hours; for ROS and gene expression, the cells were exposed for 5 hours only. Experiments were performed in triplicate, and the data reported here are the average of at least two independent experiments.

### Cytotoxicity

Propidium iodide (PI), a DNA intercalating dye that is excluded by viable cells, was used to measure cell death. Following 24 or 5 hours of exposure to PM_2.5_ extracts, cells were stained with PI and fluorescence was measured by flow cytometry (Amnis CellStream, Luminex, USA), with excitation (Ex) at 488 nm and emission (Em) at 610 nm.^28^

### ROS measurement

Cellular ROS production was measured using two different probes: 2′,7′-dichlorofluorescein diacetate (DCFH-DA) and dihydroethidium (DHE). DFCH-DA is more specific to hydrogen peroxide, while DHE preferentially measures superoxide, although both can be impacted by other oxidizing species as well.^29^ For both probes, after 5 hours of exposure to PM_2.5_ extracts, cells were incubated with 25 μM probe solution for 15–20 minutes at 37 °C and then centrifuged and resuspended in PBS. DCFH-DA fluorescence was measured at 488/529 nm (Em/Ex) and DHE fluorescence was measured at 488/620 nm (Em/Ex), both using a flow cytometer (Amnis CellStream, Luminex, USA).^28^ Within each experiment, fluorescence of cells exposed to samples was normalized to that of control cells. This normalization serves to isolate effects of samples relative to controls and account for variability in absolute fluorescence values.

### Gene expression

Total RNA was extracted from A549 cells, 5 h after exposure to PM extracts, using an RNeasy Mini Kit (QIAGEN, Hilden, Germany) and treated with RNase-free DNase to eliminate genomic DNA (Invitrogen). RNA was quantified using a NanoDrop spectrophotometer (Thermo Fisher) and 500 μg mRNA was converted to cDNA using random hexamers (Invitrogen). The expression of mRNA was quantified by real-time PCR using a Fast SYBR Green PCR mix (Applied Biosystems) with a StepOnePlus PCR instrument (Applied Biosystems). β-Actin was used as a reference gene. Primers were purchased from Sigma-Aldrich; primer sequences are listed in Table S2.† A calibration curve was run for each primer pair in order to determine the optimal cDNA dilution and verify that efficiency was close to 100% (±5%) and melt curves had a single peak.

### Data analysis

Concentrations of chemical species were blank-subtracted using an average field blank. Dust concentrations were estimated as the sum of major oxides of crustal elements (*e.g.* Al_2_O_3_ for Al) by multiplying elemental concentrations by each oxide/element mass ratio: 2.14[Si] + 1.89[Al] + 1.40[Ca] + 1.66[Mg] + 1.67[Ti].^30^ Silicon was not measured in these samples and was estimated from Al concentrations and typical crustal Si/Al ratios.^30,31^

Source apportionment was conducted using the US Environmental Protection Agency's Chemical Mass Balance Model (EPA CMB v8.2). The CMB model uses an effective variance-weighted least-squares method to estimate source contributions from organic molecular marker concentrations in source profiles and PM samples.^32^ The source profiles used were traffic emissions sampled in a tunnel in Guangzhou province,^33^ wood burning in a typical Chinese residential biomass stove,^34^ and coal combustion in a Chinese residential cooking/heating stove.^35^ Source profiles are available in Table S3.† The fitting species used in the model were levoglucosan, benzo[*b*]fluoranthene, benzo[*e*]pyrene, picene, 17α(*H*)-21β(*H*)-30-norhopane, and 17α(*H*)-21β(*H*)-hopane. *R*^2^ values were 0.9 or higher and all *χ*2 values were below 1, conforming to recommended model parameters.^36^

Statistical analysis was performed in Prism 9 (version 9.0.2, GraphPad Software) and R (version 4.0.2, http://www.r-project.org). Experimental data are presented as arithmetic means ± standard errors of the mean (SEMs). ANOVA was used to test differences in means, and individual differences were determined using Tukey HSD or Tukey–Kramer *post hoc* tests for assays with equal and unequal sample sizes, respectively.

## Results and discussion

### Chemical components and sources of PM_2.5_

Average mass concentrations of PM_2.5_ within each composite were higher in winter than in summer at all three sites. This seasonal difference was larger in the Sichuan composites (average ± standard deviation; summer: 79 ± 35, winter: 213 ± 125 μg m^−3^) than in the Beijing (summer: 73 ± 33, winter: 118 ± 65 μg m^−3^) or Shanxi composites (85 ± 29, winter: 116 ± 60 μg m^−3^). These concentrations are comparable with other PM_2.5_ exposure measurements in the study environments with household air pollution in China: in a review of PM_2.5_ exposures in rural China, Du *et al.* reported averages (within each study) of ∼100–600 μg m^−3^ in winter and ∼20–200 μg m^−3^ in other seasons,^37^ and in a recent multinational exposure study, geometric mean PM_2.5_ exposure across eleven rural sites in China was 55 μg m^−3^.^7^

Concentrations of chemical components including water-soluble organic carbon (WSOC) and ions, trace elements, and organic compounds are listed in Table 1, normalized to PM_2.5_ mass (rather than to air volume) in order to facilitate comparison between composites and with biological endpoints. The most abundant individual component was WSOC in the Beijing and Sichuan composites and sulfate in the Shanxi composites. Nitrate was notably much higher in the Beijing summer composite (39.8 μg mg^−1^) than in the other composites (up to 11.2 μg mg^−1^).

**Table 1:** Concentrations of selected chemical components, normalized to PM_2.5_ mass

	Beijing		Shanxi		Sichuan	
	Summer	Winter	Summer	Winter	Summer	Winter
**Bulk chemical composition (μg mg** ^**−1**^ **PM**_**2.5**_ **)**						
Water-soluble organic carbon	78.1	89.9	49.9	67.7	106	155
Na^+^	1.34	1.42	1.30	0.71	1.35	0.52
NH_4_^+^	73.3	47.7	33.4	50.0	27.6	18.4
K^+^	9.97	9.71	4.09	6.88	10.40	11.1
Ca^2+^	6.95	3.49	6.35	2.53	11.40	6.61
Cl^−^	2.23	11.5	0.89	10.8	4.55	6.47
SO_4_^2−^	69.9	77.7	92.2	91.3	79.8	40.7
NO_3_^−^	39.8	11.20	0	6.89	0	3.57

**Metal(loid)s (ng mg** ^**−1**^ **PM**_**2.5**_ **)**						
V	9.62	3.41	8.53	1.14	2.28	0.63
Cr	9.12	6.01	11.1	8.8	13.2	7.37
Mn	164	118	68	92.3	137	77.5
Fe	550	1620	1070	10.8	399	145
Co	0.83	0.88	1.14	0.16	0.69	1.99
Ni	21	135	49.8	0	4.23	0
Cu	70.4	52.7	32.3	0	10.7	10.9
Zn	929	707	1310	655	417	220
As	37.9	19.8	37.4	38.2	40.3	13.7
Se	16.9	27.6	26.4	57.4	13.2	14.4
Cd	13.4	12.9	8.97	17.4	13.6	11.7
Sn	12.9	0	5.34	0	6.73	0
Sb	14.8	6.75	6.02	5.17	44.7	3.56
Ba	53.2	28.7	52.8	24.4	42.1	28.60
Pb	187	219	104	420	36.6	55.7

**Organic molecular markers (ng mg** ^**−1**^ **PM**_**2.5**_ **)**						
Benzo(*b*)fluoranthene	60.8	322	66.8	434	123	79.2
Benzo(*k*)fluoranthene	41	127	18.2	84.6	109	38.4
Benzo(*j*)fluoranthene	2.56	32.9	1.41	10.7	16	11.7
Benzo(*e*)pyrene	42.2	161	69.1	372	90	37.4
Benzo(*a*)pyrene	15.1	138	11.2	86.5	81.9	49.9
Perylene	5.19	34.9	3.35	8.13	15.5	7.5
Indeno(1,2,3-*cd*)pyrene	72	230	43	217	171	84.9
Benzo(*ghi*)perylene	66.1	218	82.5	492	145	71.1
Dibenz(*ah*)anthracene	6.7	34	18.4	159	13	8.5
Picene	8	38	20.2	123	14.8	12.1
Coronene	13.4	37.9	17.4	96.9	29.5	17.4
Dibenzo(ae)pyrene	7.84	26.3	31.6	134	11.4	0
**Sum (5- & 6-ring PAHs)**	**341**	**1400**	**383**	**2220**	**820**	**418**
17α(*H*)-22,29,30-Trisnorhopane	1.41	34.5	0	4.13	0	0
17α(*H*)-21β(*H*)-30-Norhopane	3.29	66	5.5	8.79	2.32	8.95
17α(*H*)-21β(*H*)-Hopane	6.05	42.2	8.99	12.4	4.41	9.18
Phthalic acid	140	250	203	159	120	155
Isophthalic acid	16.4	25	7.8	27.8	13.1	30.8
Terephthalic acid	456	378	246	973	246	429
1,2,4-Benzenetricarboxylic acid	65.2	61.7	54.9	53.7	53	34.1
1,2,3-Benzenetricarboxylic acid	46.3	45	31.1	45.1	30.4	20.8
3-Hydroxyglutaric acid	5.92	1.82	32	0	54.9	0
2-Hydroxy-4-isopropyladipic acid	195	240	201	151	568	94
Levoglucosan	3720	4280	1240	3750	9700	8310

PAHs are formed by incomplete combustion and are emitted by vehicles, industrial, and residential combustion sources.^38^ Some are known carcinogens^39^ and in general, PAHs are thought to be important drivers of aerosol health effects.^40,41^ Total PAH concentrations were higher in winter than in summer for Beijing and Shanxi PM_2.5_, and the highest concentration of each individual PAH was also found in either Beijing winter or Shanxi winter (Table 1). Notably, in contrast to Beijing and Shanxi samples, PAH concentrations were higher in Sichuan summer than in Sichuan winter samples, although not with statistical significance.

Hopanes, which are present in vehicle lubricating oil and can be used as tracers in PM of vehicle tailpipe emissions,^42^ were higher in the Beijing winter composite (34–66 ng mg^−1^) than other composites (0–12 ng mg^−1^) (Table 1). Concentrations of levoglucosan, a product of cellulose pyrolysis that serves as a biomass burning tracer,^43^ were higher in the Sichuan composites (summer: 9700; winter: 8310 ng mg^−1^) than in Beijing (summer: 3720; winter: 4280 ng mg^−1^) or Shanxi (summer: 1240; winter: 3750 ng mg^−1^).

**Fig. 1:**
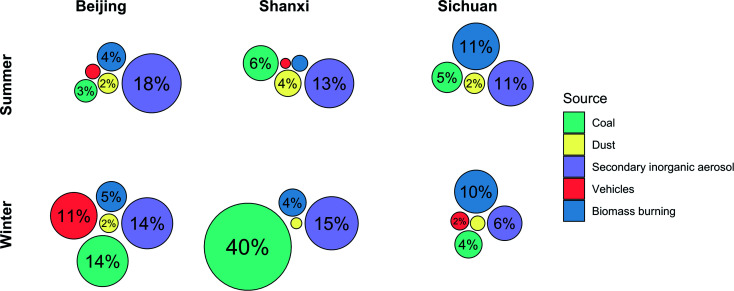
Source contributions to PM_2.5_ mass. The circular area is proportional to % contribution of each source to total PM_2.5_ mass. Unlabelled circles represent ≤1% source contribution. The order of the circles is arbitrary.

Source contributions from coal and biomass burning exhibited different trends by season and site (Fig. 1). Coal combustion comprised much more of Shanxi winter PM_2.5_ (40%) than of the other sites/seasons (3–14%), although there was also evident seasonality of coal combustion emissions in Beijing (summer: 3%; winter: 14%). Conversely, biomass source contributions did not differ by season and were the highest in Sichuan. In a previously published analysis of indoor, outdoor, and personal exposure PM_2.5_ samples from the same study site in Sichuan, biomass-burning contributions were also similar between summer and winter exposures (34% and 29% of PM_2.5_ mass, respectively), reflecting the use of biomass for both cooking and heating.^21^ Year-round biomass use may also explain why PAHs were not higher in winter in Sichuan, as they were at the other two sites. Following trends in hopane concentrations (Table 1), contributions of vehicle emissions were higher in Beijing winter (11%) than in other sites/seasons (2% or less). Dust and secondary inorganic aerosol (SIA) contributions were slightly higher in summer (dust: 2–4%; SIA: 11–18%) than in winter (dust: 2% or less; SIA: 6–15%), but generally similar between sites and seasons.

These five sources explained 30–60% of total measured PM_2.5_ mass. Secondary organic aerosol (SOA) is a heterogeneous and variable mixture that can only be identified with multivariate source apportionment techniques that require much larger sample sizes, whereas in CMB source apportionment, SOA is assumed to be part of the unapportioned mass.^44–46^ Additionally, because these are personal exposure samples, they are likely to be impacted by activity-related sources such as cooking and house dust which were not included in the CMB model, as CMB has been employed mostly for source apportionment of ambient PM.^47,48^ Although we excluded samples from participants who were currently smoking, secondhand tobacco smoke from other members of the household and community may also contribute to unapportioned mass. Additionally, most of the between-sample variances in source contributions that we observed were relatively small, which may constrain our ability to discern whether specific sources drive trends in biological endpoints.

### Cytotoxicity of water and organic extracts

**Fig. 2:**
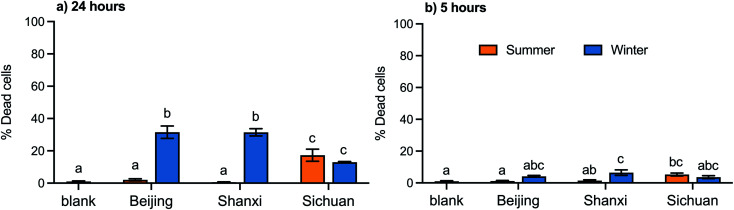
Cell death following (a) 24 hour and (b) 5 hour exposures to organic extracts (OE) (50 μg mL^−1^). Bar height and error bars represent arithmetic mean ± SEM of percent of dead cells. Means with different letters are significantly different at *p* < 0.05 (Tukey HSD test).

Cytotoxicity was measured for both water and organic extracts. The cytotoxicity of the organic extracts varied significantly by season in samples from Beijing and Shanxi, but not for Sichuan samples (Fig. 2a). Specifically, for Beijing and Shanxi, 24 h exposure to the winter organic PM_2.5_ extracts resulted in significantly higher rates of cell death (30–40%) compared with summer organic PM_2.5_ extracts (<5%). In contrast, 24 h exposure to Sichuan winter and summer organic extracts elicited similar and moderate cytotoxicity (10–20%). These cell death trends were similar to trends in total PAH concentrations (Table 1).

No significant toxicity was observed for the water extracts of any of these samples. Even at concentrations five times higher than the organic extracts (WE: 250 μg mL^−1^; OE: 50 μg mL^−1^), 24 h exposure to water extracts did not result in significant cell death from any samples (0.1–1.1%; data not shown).

Based on these cytotoxicity results, we focused on organic extracts for the remainder of the biological assays. Exposures for 5 hours, which was the duration used for all other assays, to the organic extracts at the same concentration (50 μg mL^−1^), resulted in less than 10% cell death (Fig. 2b). Under our experimental conditions, 50 μg mL^−1^ is equivalent to 4.3 μg cm^−2^, which is within the range of *in vitro* concentrations (0.2–20 μg cm^−2^) that Li *et al.* estimated to be equivalent to realistic exposures (based on measurements from a polluted area in southern California, USA).^49^

Metals and PAHs have been implicated as potential drivers of aerosol health effects, particularly *via* oxidative stress.^50,51^ Comparing water and organic solvent extracts of PM, water extracts are likely to have higher metal concentrations while PAH concentrations are higher in organic extracts.^52^ Either extract could plausibly exhibit higher cytotoxicity, and indeed, lung cell toxicity of urban PM_2.5_ has been reported to be higher for organic extracts in some instances (*e.g.* in Beijing^53^) but for water extracts in other instances (*e.g.* in Thessaloniki, Greece^54^). Other studies comparing water and organic extracts have reported different types of biological effects induced by different fractions.^26,55–57^

Most previous studies of seasonal differences in cytotoxicity of urban (outdoor) PM_2.5_ on A549 cells report that winter PM_2.5_ exposures caused more cell death or reduction in cell viability than summer PM_2.5_ exposures, including studies conducted in Beijing,^53^ Milan (Italy),^58^ Kütahya (Turkey),^59^ and Chengdu and Chongqing (southwestern China).^60^ All of these studies also reported higher winter PM levels of PAHs or organic carbon if PAHs were not measured, compared to summer PM. Cytotoxicity and/or decreased cell viability were also correlated with PAHs in studies that did not observe or investigate seasonal differences.^14,61,62^ Thus, the trends in cytotoxicity observed in this study are consistent with previous research in terms of chemical composition, if not necessarily season, demonstrating the importance of chemical analysis alongside biological assays.

### Reactive oxygen species (ROS) generation following organic extract treatment

**Fig. 3:**
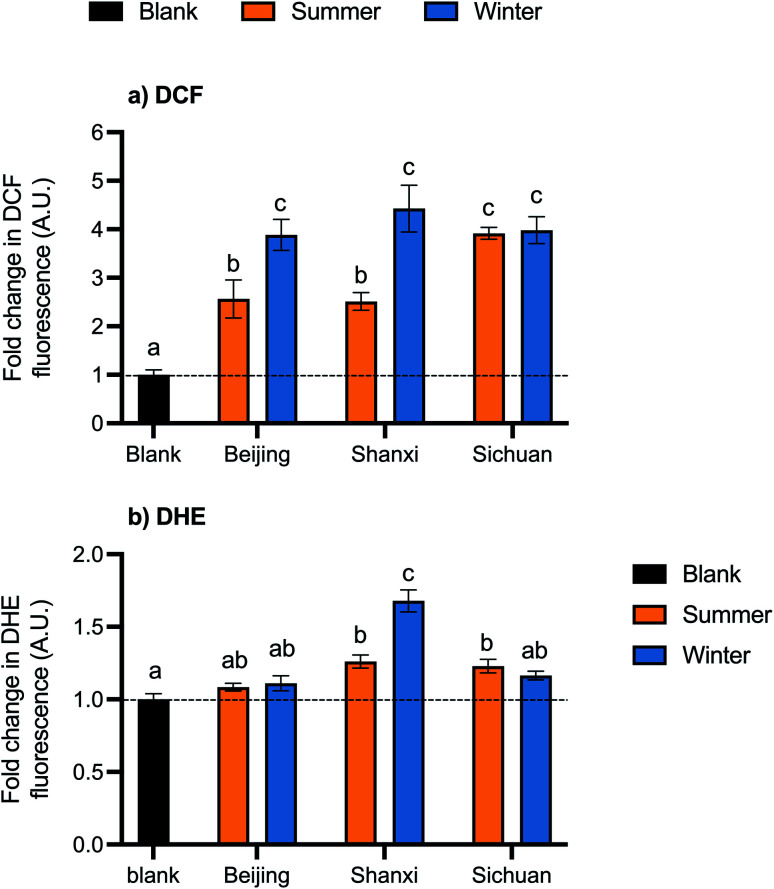
Cellular ROS generation from organic extracts, measured using (a) dichlorodihydrofluorescein (DCF) and (b) dihydroethidium (DHE) probes. Bar height and error bars represent arithmetic mean ± SEM of fluorescence normalized to controls (A.U.: arbitrary units). Means with different letters are significantly different at *p* < 0.05 (Tukey HSD test). The dashed line is plotted at *y* = 1 for reference.

Exposure to organic extracts of all samples increased cellular ROS. DCF fluorescence, which is proportional to ROS concentration, was significantly higher than the control in all samples (*p* < 0.05) by 2.6- to 4.4-fold (Fig. 3a). Between samples, DCF-ROS trends were similar to those of cell death: winter PM generated more ROS than summer PM for Beijing and Shanxi samples (average fold change in DCF fluorescence: Beijing winter 3.9, Shanxi winter 4.4, Beijing summer 2.6, and Shanxi summer 2.5). No seasonal differences were observed for Sichuan samples, however: fold changes in DCF fluorescence were 3.9 and 4.0 for Sichuan summer and winter, respectively. Although the water extracts did not exhibit any cytotoxicity in terms of cell death, this does not preclude other biological effects, so we also assessed ROS generation for the water extracts. However, exposure to the water extracts did not result in any statistically significant changes in DCF fluorescence relative to the control (Fig. S1†).

ROSs were also measured using the DHE probe, which reacts preferentially with superoxides. Exposure to the Shanxi winter sample led to the largest increase in DHE-ROS (1.7-fold) (Fig. 3b). Shanxi summer and Sichuan summer samples also had significant changes in DHE-ROS (1.3- and 1.2-fold, respectively), while Beijing summer and winter and Sichuan winter samples were not significantly different from the control. Fold changes in fluorescence were relatively smaller with the DHE probe than with DCF.

Cellular ROS levels corresponded well to measured cell death, which support the hypothesis that increased ROS production is a driver of PM toxicity due to the oxidative stress that occurs if ROS levels overwhelm the antioxidant response.^63^ Given that some PM components (*e.g.*, transition metals and quinones) are intrinsically redox-active and many more have been shown experimentally to increase ROS generation, oxidative potential may be especially relevant for component-specific toxicity.^64^

PAHs can be metabolized by cytochrome P450 enzymes, which are part of the AhR pathway, to form quinones and semiquinones that participate in redox cycling and generate additional ROS.^40^ In these samples, PAH concentrations appear to be related to ROS as measured by both probes: seasonal differences were observed in Beijing and Shanxi but not Sichuan for both PAHs and DCF-ROS, and the Shanxi winter composite had the highest total PAH content and DHE-ROS.

Additional PM components linked to toxicity include metals, which can also contribute to redox cycling *via* Fenton and Haber–Weiss reactions.^40^ Cellular ROS production and metal concentrations have been correlated in diverse sampling regions,^65,66^ and metal removal from PM extracts *via* chelation has been demonstrated to decrease cellular ROS.^50,67,68^ It is therefore somewhat surprising that water extract exposures, even at a higher concentration than organic extract exposures, did not result in a significant change in ROS levels. The exposure samples studied here have lower concentrations of metals including Fe, As, and Zn compared with the water extracts from urban PM samples that prompted ROS generation,^68^ so potentially, metal concentrations in our samples were too low to elicit a significant reaction. Additionally, many of the referenced studies showing metal and ROS associations measured cellular ROS using macrophage cells,^65,66,68^ whereas in this study we used epithelial cells, and sensitivity to metal content may differ between cell types.

### Gene expression modulation by OE treatment

To investigate the underlying mechanisms of the observed toxic effects, we used qPCR to measure changes in gene expression following exposure to organic extracts. We measured genes in three categories, broadly defined: phase I/xenobiotic metabolism (cytochrome P450 enzymes, cyp1a1 and cyp1b1; NADPH quinone oxidase, NQO-1), antioxidants and Nrf2-regulated phase II detoxifying enzymes (NQO-1; catalase; heme oxygenase, HO-1; and superoxide dismutase 1, SOD1), and inflammation (cytokines IL-6 and IL-8).

Phase I genes include some in the AhR (aryl hydrocarbon receptor) xenobiotic metabolism pathway, which can be activated by PAHs and other organic components of PM_2.5_.^69^ However, the expression of AhR-related genes was not higher in the samples with higher PAH content, *i.e.* the Shanxi and Beijing winter samples (Table 1). Expression of cyp1a1 was slightly higher than the control for all samples (1.5–1.8; *p* < 0.05 relative to the control for all except Beijing winter) but did not exhibit any seasonal or site trends; changes in cyp1b1 expression were even smaller (1.3–1.5) (Table 2).

**Table 2:** Changes in gene expression induced by organic extracts (5 h exposure, 5 μg mL^−1^)

Gene	Relative gene transcription levels^a^^,^^c^						
	Control	Beijing summer	Beijing winter	Shanxi summer	Shanxi winter	Sichuan summer	Sichuan winter
Cat	1 ± 0.05 (a)	1.1 ± 0.2 (a)	1.1 ± 0.1 (a)	1 ± 0.3 (a)	1.2 ± 0.2 (a)	1 ± 0.1 (a)	1.4 ± 0.2 (a)
**cyp1a1** b	1 ± 0.02 (a)	1.8 ± 0.2 (b)	1.5 ± 0.1 (ab)	1.6 ± 0.1 (b)	1.5 ± 0.1 (b)	1.6 ± 0.1 (b)	1.7 ± 0.1 (b)
cyp1b1	1 ± 0.05	1.3 ± 0.1	1.3 ± 0.1	1.4 ± 0.2	1.5 ± 0.1	1.3 ± 0.2	1.3 ± 0.1
HO-1	1 ± 0.1	1.8 ± 0.5	1.4 ± 0.2	1.6 ± 0.2	1.5 ± 0.3	1.1 ± 0.2	1.5 ± 0.5
**IL-6** b	1 ± 0.1 (a)	4 ± 0.6 (b)	4.5 ± 1 (b)	2.7 ± 0.5 (ab)	4.2 ± 0.8 (b)	3.6 ± 0.6 (ab)	3.8 ± 0.8 (ab)
**IL-8** b	1 ± 0.03 (a)	4.5 ± 0.8 (bcd)	6.6 ± 0.6 (d)	3.2 ± 0.6 (b)	5.5 ± 0.5 (cd)	4 ± 0.2 (bc)	4.9 ± 0.4 (bcd)
NQO-1	1 ± 0.04	0.9 ± 0.1	0.9 ± 0.04	0.9 ± 0.1	0.9 ± 0.05	0.9 ± 0.04	0.9 ± 0.03
**SOD1** b	1 ± 0.03 (a)	1.4 ± 0.1 (b)	2.1 ± 0.1 (c)	1.3 ± 0.1 (ab)	1.1 ± 0.1 (ab)	1.3 ± 0.1 (ab)	1.2 ± 0.1 (ab)
**SOD2** b	1 ± 0.01 (a)	0.8 ± 0.1 (b)	0.8 ± 0 (ab)	0.9 ± 0.1 (ab)	0.8 ± 0 (b)	0.8 ± 0 (b)	0.9 ± 0.1 (ab)

a Data are normalized to the control for each gene and presented as average ± SEM.

b Bolded gene: statistically significant ANOVA (*p* < 0.05).

c Means with different letters are significantly different at *p* < 0.05 (Tukey HSD test; shown only for genes with statistically significant ANOVA).

The Nrf2 pathway and oxidative stress in general are thought to be along the mechanistic pathway through which PM_2.5_ affects health.^40,70^ However, in these samples, we observed little change in the expression of these genes. No significant changes in expression were observed for HO-1, catalase, and NQO-1. Superoxide dismutase 1 (SOD1),^71^ which is primarily localized in the cytoplasm, was slightly upregulated in Beijing summer and winter PM_2.5_ samples (1.4 ± 0.1 and 2.1 ± 0.1, respectively; *p* < 0.05 relative to the control and each other), but not significantly by other samples (Table 2). Expression of SOD2, which is mitochondrial and expressed at lower levels than SOD1,^71^ was slightly lower than the control for all samples (0.8–0.9; *p* < 0.05 comparing between sample and control for the Beijing summer, Shanxi winter, and Sichuan summer samples).

**Fig. 4:**
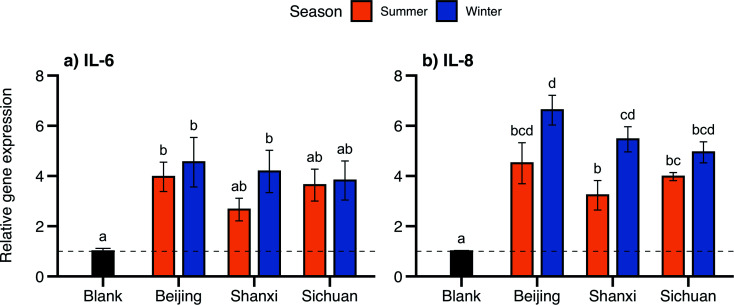
Gene expression following exposure to organic extracts for (a) IL-6 and (b) IL-8 relative to the control. Bar height and error bars represent arithmetic mean ± SEM. Means with different letters are significantly different at *p* < 0.05 (Tukey HSD test). The dashed line is plotted at *y* = 1 for reference.

Inflammation-related genes displayed the largest changes in expression: genes encoding the pro-inflammatory cytokines IL-6 and IL-8 were expressed 2.7- to 6.6-fold higher than the control for all samples (Fig. 4). Changes in IL-8 were more pronounced, with significantly higher expression for all samples, while IL-6 expression was more variable and was only significantly higher for Beijing summer/winter and Shanxi winter. IL-8 expression was higher for winter samples than their summer counterparts at each site, with larger seasonal differences for Beijing (4.5 ± 0.8 summer, 6.6 ± 0.6 winter) and Shanxi (3.2 ± 0.6 summer, 5.5 ± 0.5 winter, *p* < 0.05). Similarly, summer and winter IL-6 expression was most different for Shanxi samples (2.7 ± 0.5 and 4.2 ± 0.8, respectively), although not statistically significant (Table 2).

Exposure to samples with higher PAH content induced greater cell death and ROS production, suggesting a role of PAHs in eliciting these effects – yet, no such trends were seen in the expression of cyp1a1 and cyp1b1. Through activation of the AhR pathway, PAHs can be metabolized by phase 1 toxicant metabolizing enzymes such as cytochrome P450s, forming metabolites such as quinones that are redox-active and thus can contribute to ROS formation.^40^ Accordingly, cyp1a1 expression in lung cells proportional to PAH content of PM exposures has been reported in studies comparing PM samples with varying PAH content, such as summer and winter ambient PM.^58,72^ However, the lipophilic nature of PAHs also allows these molecules to cross cell membranes and activate pathways other than the AhR pathway.^40^ Some animal exposure studies have reported that exposure to PM extracts with higher PAH content elicits higher expression of cyp1a1 in the liver than in the lung,^52,73^ which is consistent with a model of PAH translocation beyond the lung to other organs.

Our observations of IL-6 and IL-8 upregulation following PM exposures, particularly corresponding to samples with higher PAH content, are consistent with the proposed toxicity mechanisms linking PAHs and inflammation. Systemic inflammation is likely a mechanistic driver of many adverse health outcomes associated with exposures to air pollution,^74^ and PAH concentrations were more strongly associated with inflammatory responses than overall PM mass or other chemical constituents in biomarker measurements in human epidemiological studies^5,75,76^ and both *in vivo* and *in vitro* laboratory studies.^77^ In our study samples, IL-6 and IL-8 gene expression followed similar seasonal and spatial trends as PAH concentrations, albeit with smaller differences (the summer–winter difference in gene expression was only significant for Shanxi samples and IL-8).

Although elevated ROS levels corresponding to cell death suggest that oxidative stress may be a driver of cytotoxicity induced by these samples, we did not observe significant changes in expression of oxidative stress-related genes except for SOD genes, which exhibited small and opposite changes in expression (Table 2). However, the relationship between PM exposure and expression of Nrf2/phase II protective genes is complex: exposure can elicit higher expression of these genes, but high concentrations or chronic or repeated exposures may impair cells' capacity to activate this defense system, leading to lower expression levels.^40^ Several studies reported higher Nrf2-related gene or protein expression at lower PM doses or fewer exposures than at higher PM doses or more exposures.^28,52,78,79^ Thus, not observing major effects of the samples on oxidative stress-related genes does not necessarily mean that oxidative stress is not contributing to PM-induced toxicity.

## Conclusion

Direct measurement of PM_2.5_ exposures with portable air samplers is increasingly common in epidemiological research to refine exposure assessments, but these samples are rarely studied from a toxicological perspective. Personal sampling is particularly valuable in settings with household solid fuel use, where exposures comprise a complex mixture of indoor and outdoor sources with high interpersonal variation. In this study, we assessed the *in vitro* toxicity and chemical composition of personal exposure PM_2.5_ samples collected in summer and winter from solid fuel users in three provinces in China. A central trend in both chemical composition and biological endpoints was different types of seasonal trends among the three study sites. Cell death, ROS production, and expression of pro-inflammatory cytokines were all higher in winter than in summer for the Beijing and Shanxi samples, but similar between seasons for Sichuan samples – and PAH concentrations followed the same pattern. Changes in gene expression were much larger for inflammation-related genes than for genes related to phase I xenobiotic metabolism and the AhR pathway, as well as phase II detoxifying enzymes and the Nrf2 pathway – despite the presence of PAHs, increased ROS production, and other characteristics or effects that could trigger these pathways. This complex relationship between chemical components of personal PM_2.5_ exposures and pathways of induced biological effects merits additional future research.

## Conflicts of interest

There are no conflicts to declare.

## Supplementary Material

EA-001-D1EA00022E-s001
