# Supplementary material for: Cytotoxicity and chemical composition of women's personal PM2.5 exposures from rural China
Source: Environ Sci Atmos. 2021 Jul 27;1(6):359–71. doi: 10.1039/d1ea00022e (PMC8459644; doi:10.1039/d1ea00022e)
Supplement: EA-001-D1EA00022E-s001 [file EA-001-D1EA00022E-s001.pdf]

Electronic Supplementary Material (ESI) for Environmental Science: Atmospheres.
This journal is © The Royal Society of Chemistry 2021

Supplementary Information

Table S1. Summary of filters in each composite

Site	Season	N (filters in composite)	$PM_{2.5}$ mass in composite (μg)
Beijing	summer	40	3731
Beijing	winter	35	5312
Shanxi	summer	35	3859
Shanxi	winter	30	4495
Sichuan	summer	30	6245
Sichuan	winter	20	10952

Table S2. Sequences of Homo sapiens primers used for qPCR

Gene	Forward primer (5'-3')	Reverse primer (5'-3')
β-Actin	TCGTGCGTGACATTAGGAG	CCATCTCTTGCTCGAAGTCC
IL-6	GCAAGTCTCCTCATTGAATCC	GGCACTGGCAGAAAACAACC
IL-8	TCTGGCAACCCTAGTCTGCT	AAACCAAGGCACAGTGGAAC
Cyp1a1	TCTCCTGGAGCCTCATGTATT	ACCTGCCAATCACTGTGTCTA
Cyp1b1	GAAACCTCGACTTTGCCAGG	CACCGCTTGGATTGGGATG
NQO-1	TGGTGGAGTCGGACCTCTAT	CGCAGGGTCCTTCAGTTTA
Catalase	TTCTTGGATGCAAAGTGCTG	GTTCAGCTGAACCCGATTCTC
HO-1	GAGAAAGCAAGTGGCTCACC	TGACGGACCTGGTTCTTACC
SOD1	TCACTGTGGCTGTACCAAGG	GAAAGTGCTGACCTGCATCA

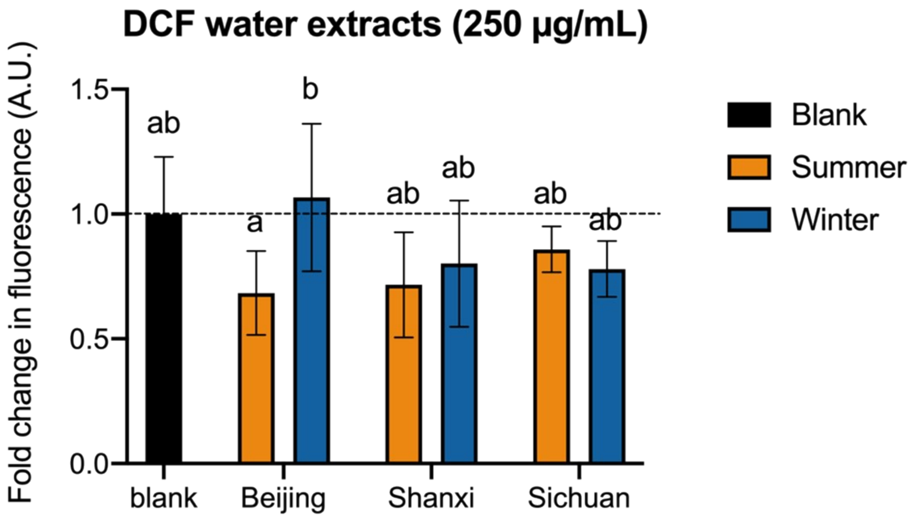

Figure S1. Cellular ROS generation from water extracts at 250 μg/mL using the dichlorodihydrofluorescein (DCF) probe. Bar height and error bars represent arithmetic mean ± SEM of fluorescence normalized to controls (A.U.: arbitrary units). Means with different letters are significantly different at $p < 0.05$ (Tukey HSD test).

Table S3. Chemical source profiles used in chemical mass balance source apportionment

	Vehicle emissions (He et al., 2008)[1]	Wood burning (Zhang, 2006)[2]	Residential coal burning (Zhang et al., 2008)[3]
Levoglucosan	0 ± 1.00E-08	8.68E-02 ± 5.92E-02	9.81E-04 ± 1.71E-03
17β(H)-21α(H)-30-norhopane	1.39E-04 ± 8.55E-06	0 ± 1.00E-08	2.19E-04 ± 3.40E-04
17α(H)-21β(H)-hopane	2.56E-04 3.27E-05	0 ± 1.00E-08	1.18E-04 ± 1.71E-04
Benzo[ghi]perylene	8.54E-05 ± 3.82E-06	3.34E-04 ± 2.72E-04	8.23E-04 ± 4.67E-04
Benzo[b]fluoranthene	4.64E-05 ± 6.09E-06	7.53E-04 ± 6.06E-04	2.18E-03 ± 1.56E-03
Benzo[k]fluoranthene	1.26E-05 ± 1.64E-06	2.26E-04 ± 1.85E-04	5.04E-04 ± 3.48E-04
Perylene	7.55E-06 ± 8.18E-07	9.12E-05 ± 7.38E-05	1.96E-04 ± 1.68E-04
Benzo[e]pyrene	3.77E-05 ± 4.09E-06	2.97E-04 ± 2.19E-04	1.12E-03 ± 6.01E-04
Picene	0 ± 1.00E-08	8.00E-06 ± 5.92E-05	2.84E-04 ± 1.59E-04
Coronene	1.65E-05 ± 1.00E-06	9.70E-05 ± 8.20E-05	1.79E-04 ± 1.14E-04
Hexacosane	1.22E-04 ± 3.27E-05	4.88E-05 ± 9.50E-07	2.62E-04 ± 4.32E-04
Heptacosane	7.08E-05 ± 1.97E-05	5.47E-05 ± 2.70E-05	1.73E-04 ± 2.65E-04
Octacosane	5.11E-05 ± 3.45E-06	1.61E-05 ± 1.90E-06	7.98E-05 ± 1.49E-04
Nonacosane	3.23E-05 ± 1.82E-06	1.76E-05 ± 4.90E-06	5.98E-05 ± 9.94E-05
Triacontane	1.63E-05 ± 2.64E-06	7.45E-06 ± 2.65E-06	3.45E-05 ± 6.58E-05
Hentriacontane	1.55E-06 ± 2.73E-07	1.71E-05 ± 1.71E-05	1.80E-05 ± 3.18E-05
Hexadecanoic acid	1.24E-04 ± 2.36E-05	1.53E-03 ± 4.40E-04	1.06E-03 ± 1.02E-03
Octadecanoic acid	5.34E-05 ± 1.40E-05	3.35E-04 ± 1.28E-04	2.06E-04 ± 2.84E-04
Eicosanoic acid	3.73E-06 ± 8.18E-07	1.25E-04 ± 5.48E-05	2.88E-05 ± 3.65E-05
